# P04 - Twenty year follow up: children prone to atopy and control children

**DOI:** 10.1186/2045-7022-4-S1-P59

**Published:** 2014-02-28

**Authors:** Liisa Kuikka, Leena Isotalo, Tiina Reijonen, Matti Korppi

**Affiliations:** 1 Helsinki University, Helsinki, Finland; 2 P-KKS, Joensuu, Finland; 3 University of Tampere, Tampere, Finland

## Introduction

Avoidance of early-life risk factors has not been successful in preventing the development of atopic symptoms /diseases and asthma.

## Methods

A prospective follow-up study for 20-years was conducted in Kuopio, Finland starting the year 1981 (N=152). Intervention measures included the avoidance of house dust mite, pet dander allergens and passive tobacco smoking. Prolonged breast feeding was encouraged with formula supplementation if necessary, and introduction of solid foods was delayed until four months or later.

## Results

At the age 20 years, atopic eczema was equally common despite of atopic heredity. Allergic rhinitis was significantly (p=0.004) more common in children prone to atopy. Atopic eczema at age 2, 7 and 18 years flagged atopic eczema lasting until young adulthood, and predicted later rhino conjunctivitis (60%) and asthma at age 20y (28%). Timothy grass (65%) and birch pollen (55%) sensitization were the two common responses to findings in skin prick tests.

Heredity was the most important cause for later allergic disease. At age 20 years, howeve, the male gender was the most important factor for birch pollen allergy connected with atopic eczema (p=0.01) or allergic rhino conjunctivitis (p=0.04).

## Conclusion

We found no effect of environmental or nutritional factors for later allergic diseases. Heredity and early atopic signs flag for later atopic diseases.

